# Case Report: Endoscopic Sistrunk and thyroidectomy via the areola approach in patients with thyroglossal duct cysts and thyroid cancer

**DOI:** 10.3389/fonc.2026.1618501

**Published:** 2026-03-02

**Authors:** Jinyan Hu, Zilai Hu, Jia Li, Mimi Shen, Suansuan Zhu, Lanhai Zhang, Wei Han, Yi Luo, Leilei Li, Xiaoyu Fang, Songjiang Liu, Junyuan Lv, Taolang Li

**Affiliations:** 1 Department of General Surgery, Affiliated Hospital of Zunyi Medical University, Zunyi, China; 2 Department of Thyroid and Breast Surgery, Affiliated Hospital of Zunyi Medical University, Zunyi, China; 3 Department of Radiology, Affiliated Hospital of Zunyi Medical University, Zunyi, China

**Keywords:** endoscopy, Sistrunk, thyroglossal duct cyst, thyroid cancer, thyroidectomy

## Abstract

Thyroglossal duct cyst (TGDC) is the most prevalent congenital cervical anomaly. As the standard of care, Sistrunk operation via transverse cervicotomy is used to surgically treat TGDC. Over the last four decades, prevalence of thyroid cancer has increased, making it the most common endocrine malignancy worldwide. The standard surgical method is open thyroidectomy, which is typically conducted through a cervical incision. However, the co-occurrence of TGDC and thyroid cancer is extremely rare, and there are currently no standard clinical guidelines for managing both conditions simultaneously. Endoscopic approaches have become increasingly popular for neck surgery because of improved postoperative aesthetics and recent advances in endoscopic methods. We therefore report a novel technique for endoscopic Sistrunk surgery and thyroidectomy via the areola approach in a 24-year-old female patient with both TGDC and papillary thyroid cancer (PTC). The patient recovered well and was discharged on the second day after surgery. To our knowledge, this is the first report of simultaneous endoscopic treatment of TGDC and PTC via the areola approach. Our results demonstrate that endoscopic resection and thyroidectomy via the areola approach is an effective surgical modality and a viable alternative for the complete removal of TGDC and PTC without neck scarring, especially for young patients who wish to obtain better cosmetic results.

## Introduction

Thyroglossal duct cyst (TGDC) is the most prevalent congenital cervical anomaly (1) and a type of soft tissue cyst commonly found in children and adolescents (2). Continuity with the tongue can lead to infection by oral bacteria, which may transform into an abscess and eventually cause intermittent drainage via a fistula opening (3). As the standard of care, the TGDC is surgically treated with a Sistrunk operation via transverse cervicotomy (4). This includes the removal of the hyoid body, part of the basal lingual muscle and the basal lingual mucosal ring adjacent to the blind hole. This procedure, first described by Sistrunk in 1920, has been shown to reduce the recurrence rate of TGDC to 1.3–2% (5).

Relatedly, thyroid cancer has become more prevalent over the last four decades, becoming the most common endocrine malignancy worldwide (6). The most common subtype of thyroid cancer, namely, papillary thyroid cancer, has the best overall prognosis. The lymph nodes in the cervical region are most commonly affected by metastasis, whereas the lungs are affected less frequently (7). The standard method is open thyroidectomy, which is typically conducted through a cervical incision. Traditional open thyroidectomy involves a 5 to 10-cm incision in the neck, which has a negative aesthetic impact, especially in young patients, and may lead to psychological trauma and a reduced quality of life (8).

By minimizing visible neck scarring, the endoscopic approach can improve cosmetic outcomes. It is increasingly used for thyroidectomy, parotidectomy, and cervical lymph node dissection (9).

Despite the increasing use of endoscopic technology in TGDC and PTC, it is extremely rare for these two conditions to occur simultaneously. To date, only a few cases of simultaneous TGDC and PTC have been reported in the English literature, and all of these cases were surgically treated. To our knowledge, this is the first report of simultaneous endoscopic administration of TGDC and PTC via the areola approach. Hyung Kwon Byeon has previously reported robot-assisted Sistrunk surgery, total thyroidectomy, and neck dissection via the Transaxillary and Retroauricular (TARA) approach for TGDC and PTC. By sharing our experience, we aim to provide a valuable reference for minimally invasive treatments for this rare condition.

## Case

**Figure 1:**
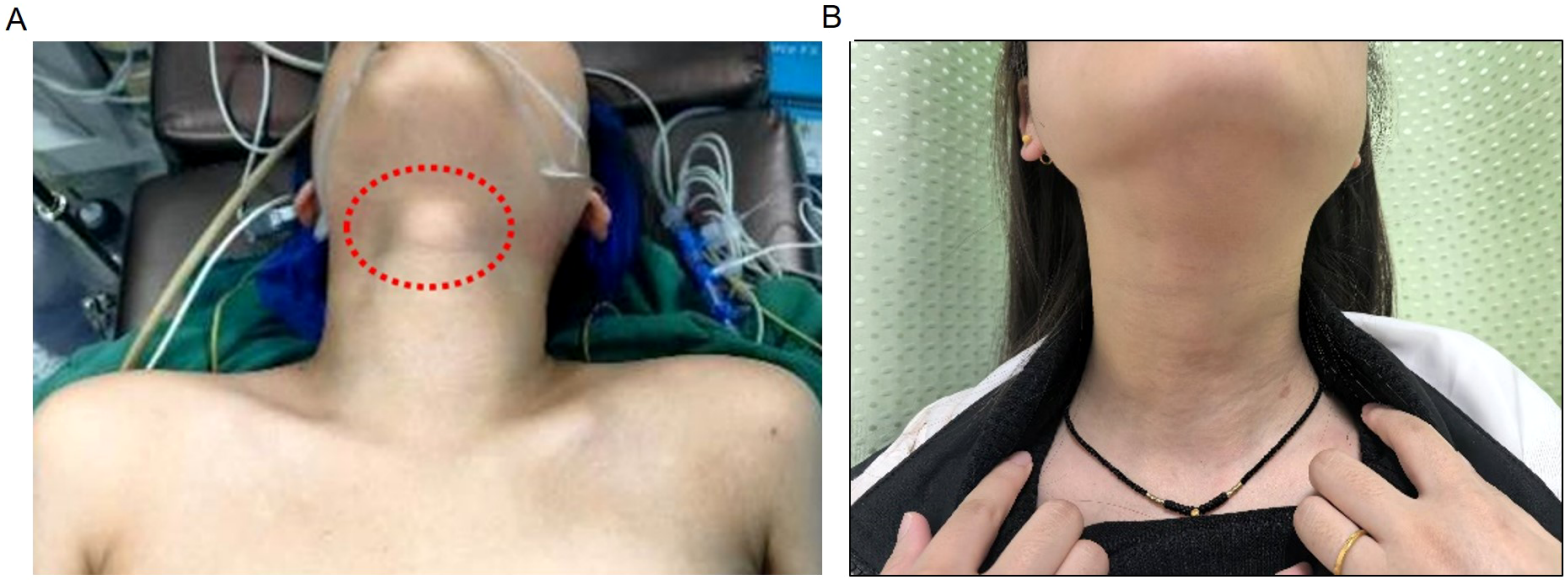
Comparative pre- and post-operative photographs of the cervical mass are provided. **(A)** Physical examination revealed a mass about 2×2 cm in size above the laryngeal prominence, which was firm in texture, had a clear boundary, was immobile and non-tender. A tumor about 2×2 cm in size was palpated in the left thyroid region, which was hard in texture, had an unclear boundary and moved up and down with swallowing. **(B)** Five months after the operation, there was no lump in the neck.

A 24-year-old female patient presented to the Affiliated Hospital of Zunyi Medical University with a thyroglossal duct cyst and thyroid nodules that were detected during a physical examination four years prior. On physical examination, a single location of painless swelling measuring 2 × 2 cm was palpated under the hyoid bone in the midline of the neck, and a painless mass measuring 2 × 2 cm and with indistinct boundaries, was palpated in the left lobe of the thyroid (**Figure 1A**). There was no palpable cervical lymphadenopathy. The patient had no medical history, and the results of the remaining systematic review were unremarkable.

**Figure 2:**
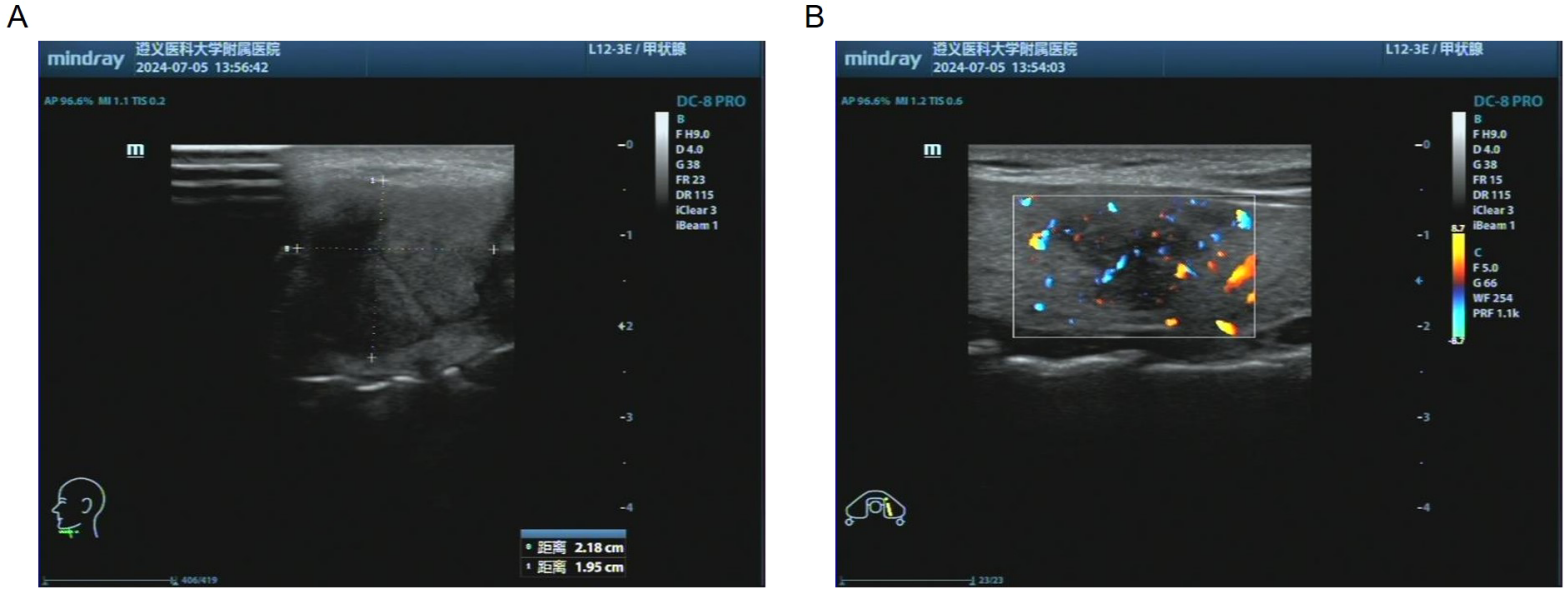
Results of preoperative cervical ultrasonography. **(A)** Ultrasonography revealed the hypoechoic area in the anterior cervical region, measuring 22×20 mm. **(B)** A 20×20 mm hypoechoic area of the left lobe thyroid was irregular in shape, ill-defined in boundary, and heterogeneous in internal echo.

**Figure 3:**
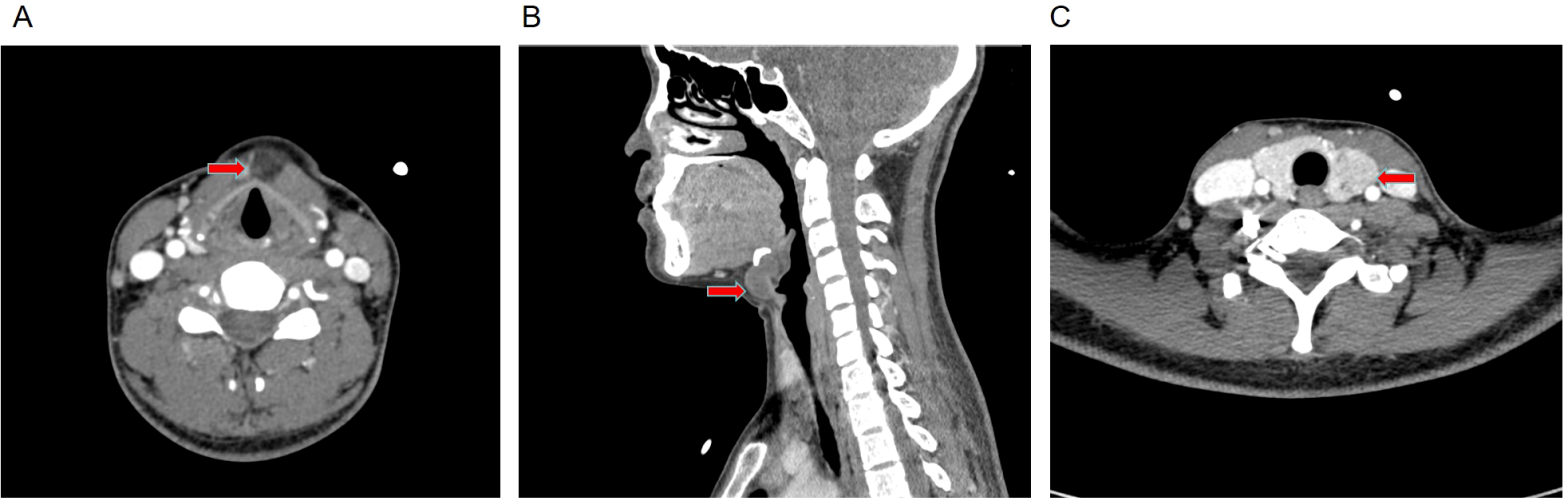
Computed tomography findings of the cervical mass. **(A)** Low-density cystic cavity is present in the hyoid cartilage-thyroid cartilage space, measuring 12×18×22 mm, with a CT value of 25 HU. **(B)** The boundary is clear and there is no enhancement on enhanced scanning. **(C)** An irregular low-density mass of 20×20×30mm in size was found in the left lobe of the thyroid gland. The boundary was unclear. The enhanced scan showed significant uneven enhancement, and the CT value was still lower than that of the normal thyroid gland.

Ultrasonography revealed the hypoechoic area in the anterior cervical region, measuring 22 × 20 mm. A 20×20 mm hypoechoic area of the left lobe thyroid was irregular in shape, ill-defined in boundary, and heterogeneous in internal echo (**Figures 2A, B**). The typical computed tomography findings of TGDC are circular cystic nodules at or near the midline. In this case, computed tomography revealed a cystic low-density shadow measuring 12×18×22 mm in the thyroid-hyoid space with poorly defined borders and a CT value of 25HU. The boundary was clear, and there was no enhancement on enhanced scana. The irregular low-density mass of the left lobe of the thyroid, 20×20×30 mm in size, with unclear boundaries, marked uneven enhancement on enhanced scan, and CT values were still lower than normal thyroid. (**Figures 3A–C**).

The most common mutated gene in nonmedullary thyroid cancer is BRAFT1799A, resulting in a BRAFVE-mutant kinase that is present only in papillary thyroid cancer (10). In this case, fine-needle aspiration cytology (FNAC) revealed papillary thyroid cancer of the left lobe (TBSRTC VI), and the BRAFV600E gene mutation was detected.

After the patient was anesthetized, the shoulder was elevated and the head was tilted back, with the neck in a hyperextended position. Subcutaneous tunnels were established through the areola approach (two 1-cm incisions on the outer edge of the right areola and one 1-cm incision on the left areola) (**Supplementary Figures 1A, B**). Then, the neck flap was separated under direct vision to create an operating space (**Supplementary Figures 1C, D**). Upon exposure, the thyroglossal duct cyst was cystic and pliable, exhibiting a smooth, sharply demarcated border with only minor local adhesions (**Supplementary Figures 1E, F**). First, the TGDC was separated (**Supplementary Figures 2A–C**), the root of the cyst was located, and the cyst was resected after clamping the vessels below the hyoid bone (**Supplementary Figures 2D–F**). Following further mobilization of the thyroid lobe, carbon nanoparticles was selectively infiltrated into the parenchyma to delineate the lymphatic channels and provide negative mapping of the parathyroid glands (**Supplementary Figures 3A, B**). A solid, well-circumscribed nodule with a smooth surface was identified within the left thyroid lobe; the lesion did not breach the thyroid capsule. After confirming tracheal position, the thyroid isthmus was transected; the left lobe was elevated and serial extracapsular dissection was performed along the outer margin of the mass (**Supplementary Figures 3D–F**). Subsequently, a total left thyroidectomy and left level VI lymph node dissection were performed and a closed-suction drain was placed. The left recurrent laryngeal nerve was meticulously exposed and safeguarded throughout, and both superior and inferior parathyroid glands were preserved (**Supplementary Figures 4A–F**, **5A–D**). The linea alba cervicalis was re-approximated along the midline and a drain was placed (**Supplementary Figures 5E, F**), the cervical incision was closed in layers, and the areolar skin portals were sutured, completing the operation. Hemostasis was scrupulous, and post-operative serum calcium and parathyroid hormone levels remained within normal limits (View video). The operation took approximately 1 hour and 40 minutes, with an estimated blood loss of 6 mL. Intraoperative visualization was provided by a Karl Storz ENDOSKOPE TC200 system, and tissue division was performed with a high-frequency electrosurgical unit set at 420X.

**Figure 4:**
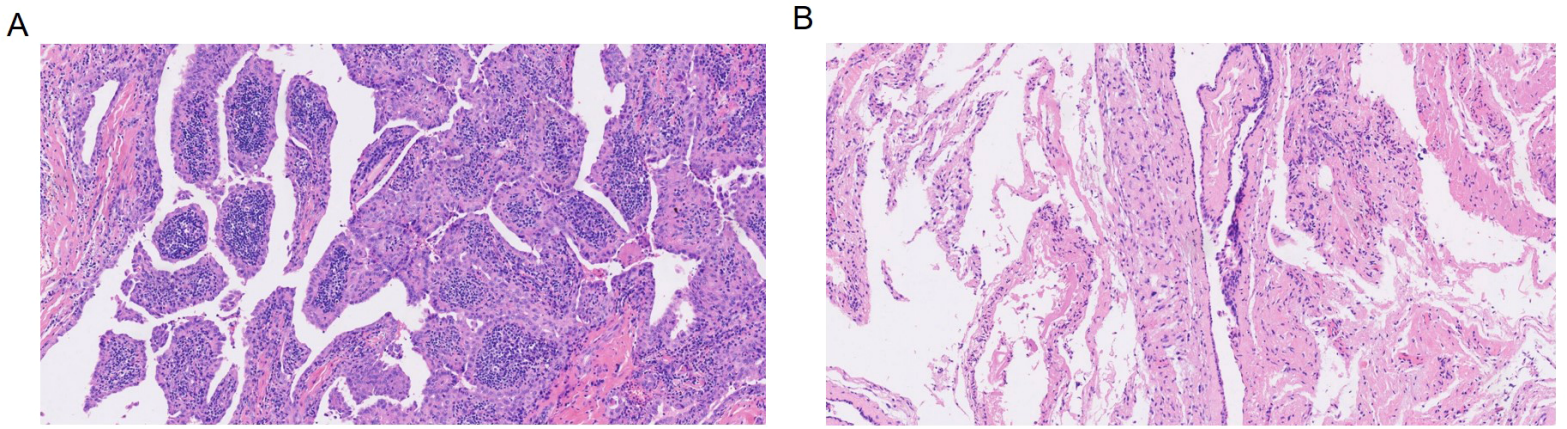
Postoperative histopathology images (H&E staining, ×10 magnification). **(A)** Left papillary thyroid cancer. **(B)** Thyroglossal duct cysts (Thyrohyoid bone).

**Figure 5:**
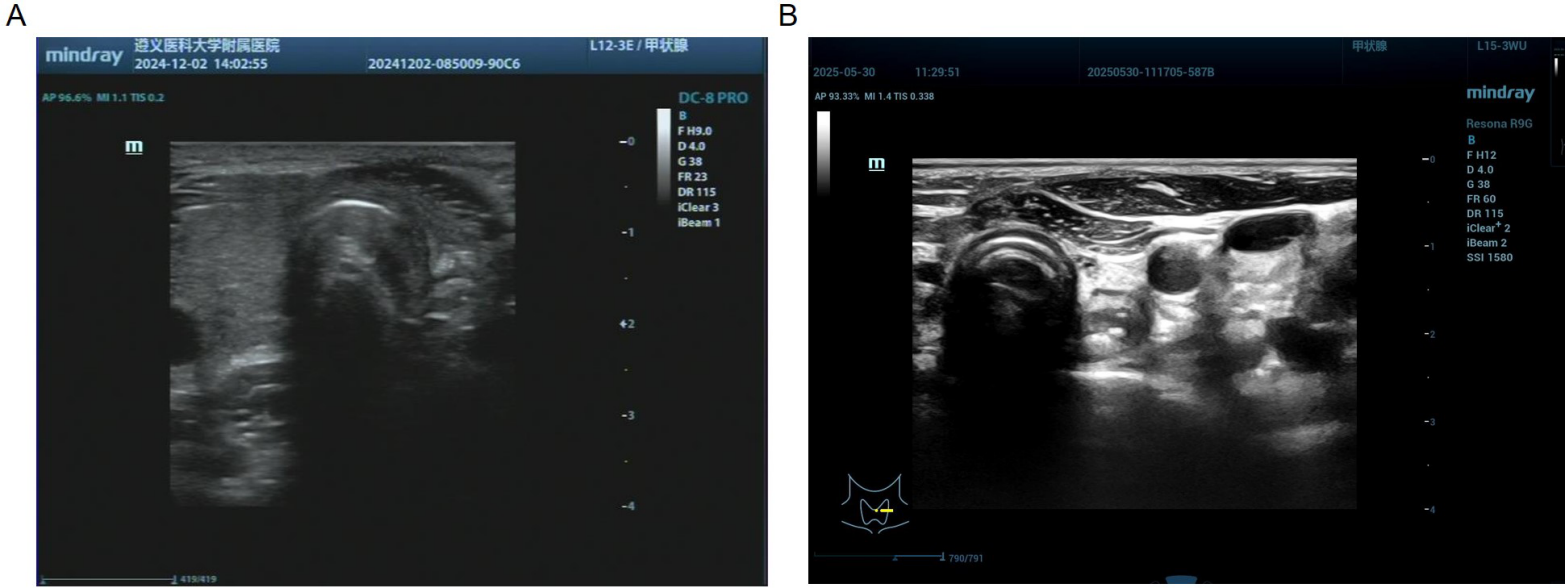
Results of Postoperative follow-up ultrasonography. **(A)** Ultrasonography performed on December 2, 2024, showed the left thyroid bed demonstrated absence of any residual glandular or nodular echoes. **(B)** Ultrasonography performed on May 30, 2025, still showed no recurrence.

Postoperative histopathology revealed left papillary thyroid cancer, left central lymph node metastasis (1/9), and thyroglossal duct cysts(Thyrohyoid bone) (**Figures 4A, B**). Laboratory tests revealed that Ca was 2.26 mmol/L and that PTH was 31.0 pg/ml. The patient recovered well, with no hematoma in the operation area, no numbness to the hands or feet, and no hoarseness or other discomfort. Therefore, the patient was discharged on the second day after surgery. At the 1-month visit, laboratory evaluation revealed thyroglobulin antibody 236 IU/mL, T3 7.5 pmol/L, and TSH 0.036 μIU/mL. Five months later, there was no mass in the neck on physical examination, and the patient felt slight numbness in the local skin, and T3, T4, TSH, thyroglobulin, TPOAb and TgAb were controlled at a reasonable level. (**Figure 1B**). Ultrasonography of the left thyroid bed demonstrated absence of any residual glandular or nodular echoes (**Figure 5A**). One year postoperatively, the patient remained free of cervical mass, with serum T3, T4, TSH, thyroglobulin, TPOAb, and TgAb all within normal limits. Ultrasonography still showed no recurrence (**Figure 5B**), and we are continuing to monitor the patient with no evidence of recurrence to date.

## Discussion

Anatomically, the TGDC starts at the blind foramen of the tongue, runs forward and below the hyoid bone, then reaches the hyoid bone to form a C shape to enclose the hyoid bone, and then moves down to connect with the thyroid gland. Furthermore, TGDCs occur in approximately 7% of adults and typically presents as a painless mass in the midline of the neck, often located just below the hyoid bone (11). TGDC is capable of movement with swallowing and tongue extension and can take place anywhere from the blind lingual foramen to the suprasternal notch (12).

After the diagnosis of TGDC, surgery should be performed before infection occurs. Treatment of the cervical TGDC is usually performed by means of the Sistrunk operation, which was first described in the 1920s. Notably, this procedure reduces the recurrence rate of TGDC from 50% to 2–6% (13). The criteria for the extent of resection in the Sistrunk procedure include the removal of TGDCs within the middle segment of the hyoid bone and supraglottic muscle tissue from the blind orifice of the tongue mucosa (14). Since the majority of patients who undergo surgery are young, the standard transcervical approach requires an incision in the neck, inevitably leading to scarring, which is unsatisfactory in terms of the cosmetic outcome (15). To avoid the appearance of neck scarring, several related endoscopic Sistrunk procedures have been pioneered and gradually applied in clinical treatment. These surgical approaches include the bilateral axillary-breast approach, oral vestibular approach, transoral fasciotomy approach, retroauricular approach and bilateral areola approach (16). Although these approaches have demonstrated safety, feasibility, and favorable cosmetic outcomes, each carries inherent drawbacks. The retroauricular incision, though hidden by hair, may occasionally develop hypertrophic scarring and reveal the surgical history. The ransoral vestibular approach requires a sublingual mucosal incision that can injure floor-of-mouth structures, resulting in transient dysphagia or dysarthria. Both retroauricular and transoral routes also offer limited working space, restricting instrument manipulation and increasing operative complexity (16). Moreover, published data indicate a higher infection rate with transoral access (17). Endoscopic Sistrunk operation via the areola approach is a safe and available method for TGDC resection. This method has the advantages of good cosmetic results and does not produce scarring in the neck, providing a new strategy for the pursuit of the best cosmetic results (18). A scoping review revealed that the operative duration of endoscopic Sistrunk procedures varied from 50 to 480 min across series. Seven patients (8%) experienced complications such as infection, skin bruising, and dysarthria. No conversions to open surgery or post-operative recurrences were documented (9). By contrast, our endoscopic procedure was completed in 1 h 40 min while simultaneously accomplishing total thyroidectomy and cervical lymph-node dissection.

Differentiated thyroid cancer, which originates from the follicular epithelial cells of the thyroid, accounts for more than 95% of thyroid cancers in terms of incidence and is the most common subtype of thyroid cancer (19). For malignant thyroid diseases and benign diseases that cannot be treated with drugs, thyroidectomy is the preferred treatment. The most common complications after thyroidectomy are hypoparathyroidism and hypocalcemia, usually due to accidental injury or removal of the parathyroid gland during surgery (20). Studies have shown that total endoscopic thyroidectomy and conventional open thyroidectomy are similar in the evaluation of thyroid cancer in terms of safety and efficacy, with no significant differences in tumor recurrence rates or surgical integrity. Indeed, total endoscopic thyroidectomy results in markedly lower levels of transient hypocalcemia and better cosmetic outcomes; thus, it is a better choice for patients with cosmetic hesitations (21). The breast approach has been one of the most widely used among the various approaches to thyroidectomy (22). For patients with stage T1 papillary thyroid cancer, endoscopic thyroidectomy via the areolar approach is a safe and feasible surgical approach that is similar to conventional open thyroidectomy in terms of tumor resection integrity while avoiding neck scarring and obtaining excellent cosmetic results (23). Although minimally invasive thyroidectomy provides better cosmetic results, it also has corresponding limitations, mainly including prolonged operation time, increased incidence of transient recurrent laryngeal nerve paralysis, and a larger gap during flap retraction. Furthermore, it requires a significantly longer learning time and higher cost (24). Therefore, as an alternative option, endoscopy should be strictly considered in clinical application and implemented after individual evaluation.

In this case, TGDC resection and left lobe thyroidectomy with isthmus resection was performed, and the left central lymph node was removed. The co-occurrence of TGDC and thyroid cancer is particularly rare, and there are currently no standard clinical treatment guidelines. However, there have been similar reports. Q. Cai’s research team used endoscopy to simultaneously remove TGDCs and benign thyroid nodules through the submental approach for 6 patients with TGDC and benign thyroid nodules, and the operation was successfully completed (25). There are also reports of thyroglossal duct carcinoma, and one of the surgical methods includes the combined operation of the Sistrunk operation and thyroidectomy (26). For combined surgery, an open incision is usually used to remove the TGDC and thyroid cancer with or without cervical lymph node dissection (27–33). However, there was one case of ectopic thyroid carcinoma in the TGDC in which a second thyroidectomy was performed after the Sistrunk operation (34). Hyung Kwon Byeon presented a case of combined surgical procedures, robot-assisted Sistrunk operation, total thyroidectomy, and cervical neck dissection for TGDC carcinoma and thyroid cancer via the transaxillary and retroauricular approaches. This combined procedure was performed by means of two approaches, and disease treatment was completed (35).

Considering that the patient was a young female with a strong aesthetic need to avoid scarring on the front of the neck, the patient’s medical history was comprehensively evaluated. Among the major risk factors for TGDC recurrence, the most decisive risk factor is a history of preoperative TGDC infection (36). TGDCs have clear boundaries, and no infection or abscess is formed. Additionally, TGDCs can be removed via an endoscopic transthoracic breast approach. For T1 papillary thyroid cancer with a diameter of ≤ 2 cm endoscopic thyroidectomy via the areola approach is indicated. A meta-analysis (37) indicates that endoscopic total thyroidectomy achieves surgical outcomes comparable to open surgery, while different endoscopic techniques demonstrate specific strengths across various endpoints. Specifically, the transoral approach shows an advantage in lymph node retrieval. The minimally invasive video-assisted technique excels in controlling operative time, intraoperative blood loss, and the rate of permanent hypoparathyroidism. The endoscopic gasless transaxillary approach effectively shortens hospital stay and reduces the incidence of transient hypoparathyroidism. Furthermore, the endoscopic bilateral areola approach offers superior protection for the recurrent laryngeal nerve. In a study (22), the transareolar approach facilitated visualization of the central lymph nodes, while the gasless transaxillary approach, with its lateral viewing angle, likewise enabled thorough clearance of the central compartment and provided clear exposure of the carotid artery, ensuring comprehensive dissection. Both endoscopic approaches demonstrated significantly better cosmetic satisfaction compared with the open surgery group, with no statistically significant difference observed between the two endoscopic methods. Additionally, there was no significant difference in postoperative parathyroid hormone (PTH) levels between either endoscopic group and the open surgery group. The transaxillary approach, as its dissection proceeds within natural cervical planes without disruption of the linea alba cervicis, can minimize postoperative swallowing discomfort and prevent adhesions between the strap muscles and the platysma flap. However, this technique is more demanding, with an average operative time of 125 minutes, which is longer than that of open surgery. Another study comparing the transoral and transareolar approaches found that the transoral approach required a longer operative time. No significant differences were found between the two approaches in terms of blood loss, total lymph node yield, length of hospital stay, or the incidence of transient hoarseness (38). Nonetheless, multiple studies have demonstrated that the transoral approach does not compromise safety or oncologic outcomes compared to the transareolar approach, while providing superior cosmetic results and enhanced quality of life (39–42). The primary benefit of the retroauricular approach is its excellent scar concealment, as incisions are hidden by the hairline or attire. Notable considerations include potentially restricted instrument angles and its relatively specialized application, making it most appropriate for select patients. More large-scale, controlled studies remain essential for a thorough assessment of surgical outcomes and long-term prognosis. Clinicians should integrate a comprehensive evaluation of the patient’s status with their preferences to tailor the surgical approach.

TGDC is characterized by an active painless mass in the midline or slightly lateral neck and is located below the hyoid bone in approximately 75% of patients (43). The normal thyroid is located in the subhyoid splanchnic space of the neck (44). The two lesions are adjacent to each other and can be resected by endoscopic surgery at the same time to avoid secondary surgery and redundant incisions. On the basis of previous surgical experience, we developed a novel surgical approach to meet the cosmetic and psychological needs of our patients, with maximum consideration of cosmetic effects while achieving therapeutic benefits. The patient was very satisfied with the results of the operation. Importantly, the recurrent laryngeal nerve and parathyroid gland were effectively protected during the operation, and there was no conversion to open surgery or intraoperative complications. The patient recovered well postoperatively and did not exhibit hypocalcemia, parathyroid function impairment, difficulty swallowing or other postoperative complications.

The endoscopic transaxillary approach provides enhanced exposure for the dissection of the superior thyroid pole, Berry ligament, and the external branch of the superior laryngeal nerve, facilitates lymphadenectomy, and achieves superior cosmetic results. However, access to the caudal central compartment is compromised by the clavicle and sternum, which obstruct the endoscopic view and impede instrument movement (45). Its application in male patients or females with smaller breast development warrants cautious evaluation due to this inherent limitations. Male sex is an independent risk factor for conversion to open surgery (46). In male patients, the typically smaller areola diameter and more developed pectoralis major muscle can restrict the operational space and suboptimal instrument angles, potentially increasing the technical difficulty of procedures such as central compartment lymph node dissection. Conversely, in females with smaller breasts, the limited volume of subcutaneous tissue and breast parenchyma may compromise the stability of the created operative cavity. This increases the risks of thermal injury to the skin and postoperative breast asymmetry. Therefore, a detailed preoperative anatomical assessment is paramount. Alternative approaches, such as the transaxillary route, should be considered when anatomical constraints are anticipated. The bilateral areola approach is best indicated for patients with adequate breast volume and a strong desire for a scarless neck.

This report is fundamentally limited by its nature as a single-case report. Although it presents an innovative surgical strategy for the rare co-occurrence of TGDC and PTC, the design carries an inherent risk of selection bias and precludes reliable estimation of long-term oncology prognosis, complications, and recurrence due to the absence of a control group. Ultimately, confirming the oncological safety and generalizability of this combined technique necessitates prospective, multicenter trials incorporating predefined eligibility criteria, standardized surgical procedures, and prolonged follow-up. Furthermore, we propose long-term oncological surveillance recommendations. Given that the patient has both TGDC and PTC and has undergone thyroidectomy, we advise following the long-term monitoring guidelines for differentiated thyroid cancer, along with local follow-up for the TGDC resection. This includes postoperative PTH assessment and measurement of serum thyroglobulin (Tg) and Tg antibodies every 6–12 months, which are highly sensitive indicators for detecting recurrence or metastasis. A neck ultrasound should be performed within the first year after surgery to evaluate the thyroid bed and cervical lymph nodes. Subsequently, depending on the risk of recurrence, imaging may be repeated every 1–2 years. For high-risk patients, diagnostic whole-body radioactive iodine scanning may be considered when necessary. Based on the initial tumor stage and recurrence risk stratification, TSH should be maintained within a target range of 0.5–2.0 mU/L to reduce recurrence risk while minimizing medication side effects. Although malignant transformation of TGDC is rare and complete excision has been achieved, it is still recommended to monitor the hyoid region during periodic neck ultrasounds for any abnormal recurrent masses.

## Conclusion

The co-occurrence of TGDC and PTC is extremely rare and presents unique challenges for surgeons. We report the first case of simultaneous TGDC and PTC excision by means of Sistrunk endoscopic surgery and thyroidectomy with the areola approach for this condition. This new method allows two lesions to be removed simultaneously through the same incision, avoiding visible neck scars and providing excellent cosmetic results. Our results demonstrate the feasibility, safety, and efficacy of this approach in young female patients with coexisting TGDC and PTC. We believe that this method is a promising option for specific patients who prioritize cosmetic results and wish to avoid cervical scarring. Further studies are needed to validate the oncological outcomes and long-term benefits of this approach in larger patient cohorts. Careful patient selection, preoperative planning, and advanced endoscopic skills are essential to ensure the success and safety of this procedure.

## Data Availability

The original contributions presented in the study are included in the article/**Supplementary Material**. Further inquiries can be directed to the corresponding authors.
