# Supplementary figures and images for: Case Report: Endoscopic Sistrunk and thyroidectomy via the areola approach in patients with thyroglossal duct cysts and thyroid cancer
Source: Front Oncol. 2026 Mar 2;16:1618501. doi: 10.3389/fonc.2026.1618501 (PMC12989352; doi:10.3389/fonc.2026.1618501)

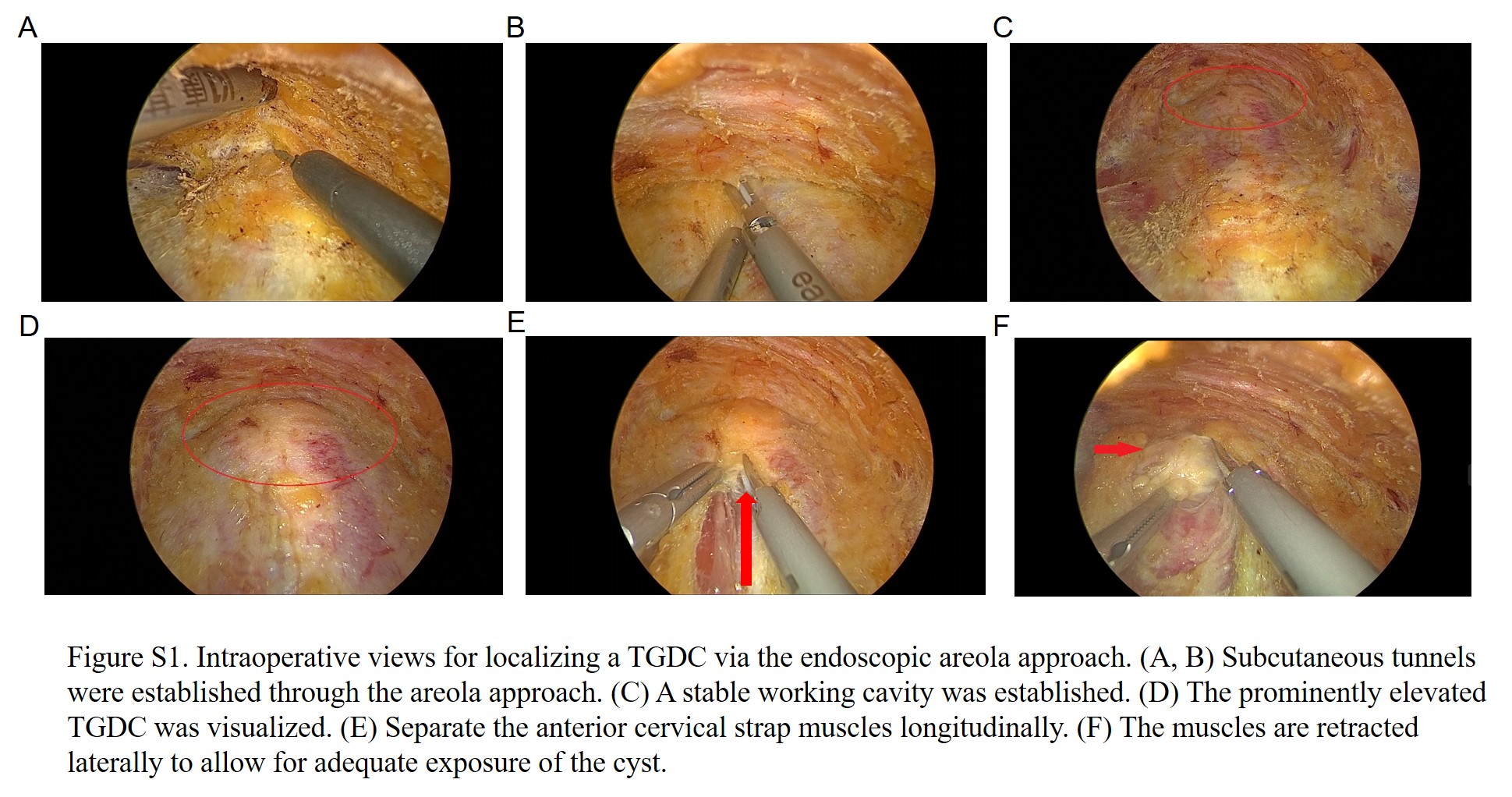

Supplement: Supplementary file 1 [file Image1.jpeg]

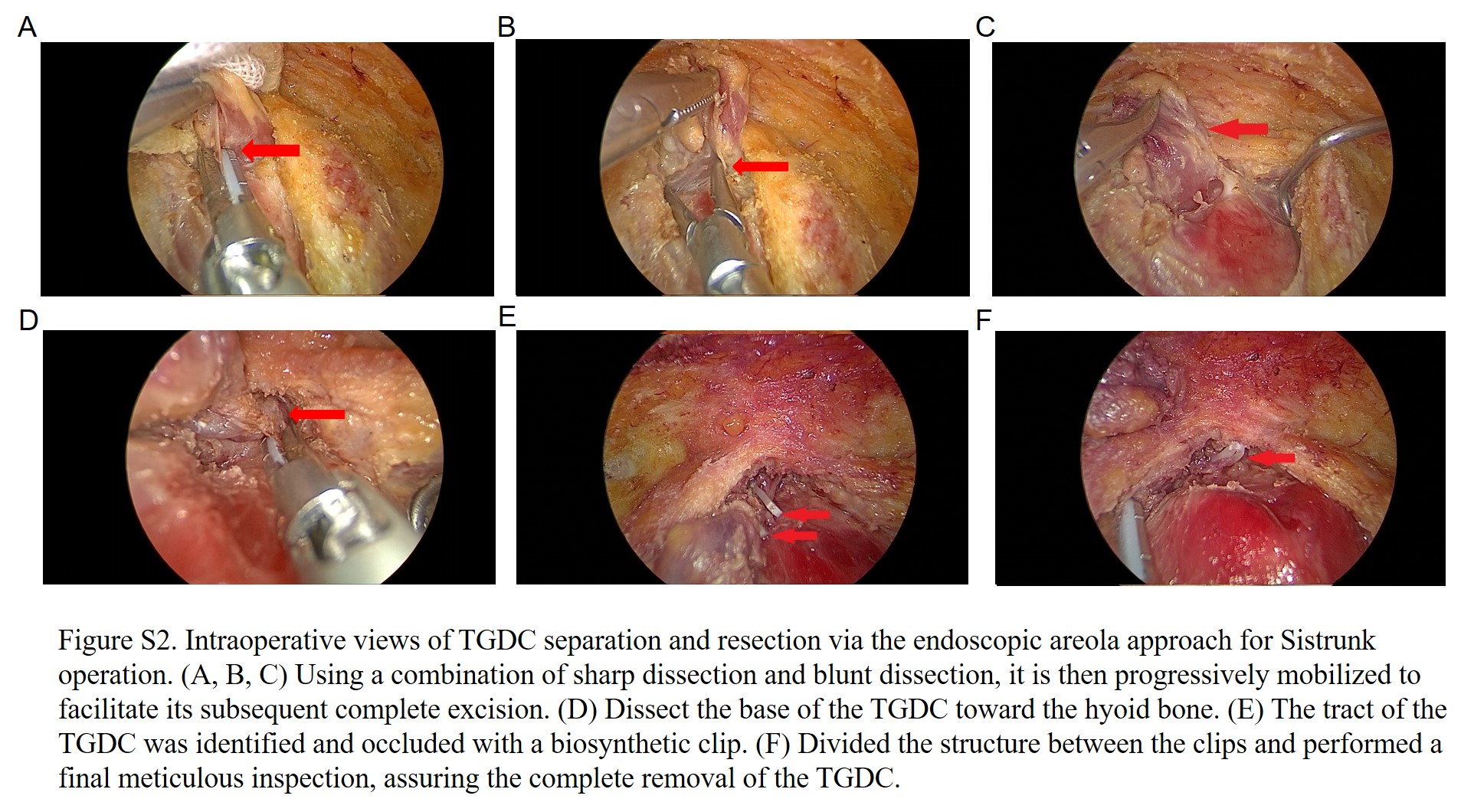

Supplement: Supplementary file 2 [file Image2.jpeg]

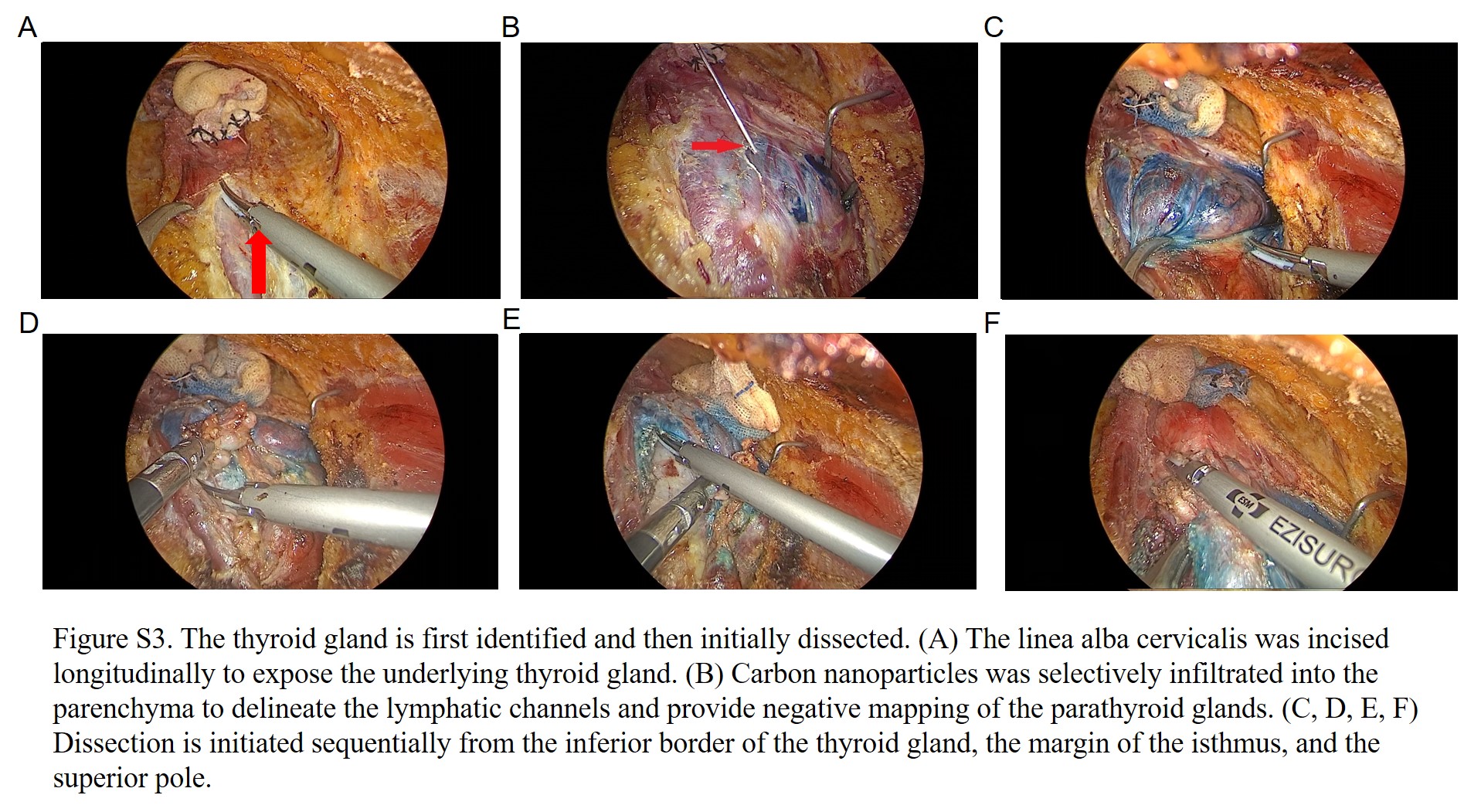

Supplement: Supplementary file 3 [file Image3.jpeg]

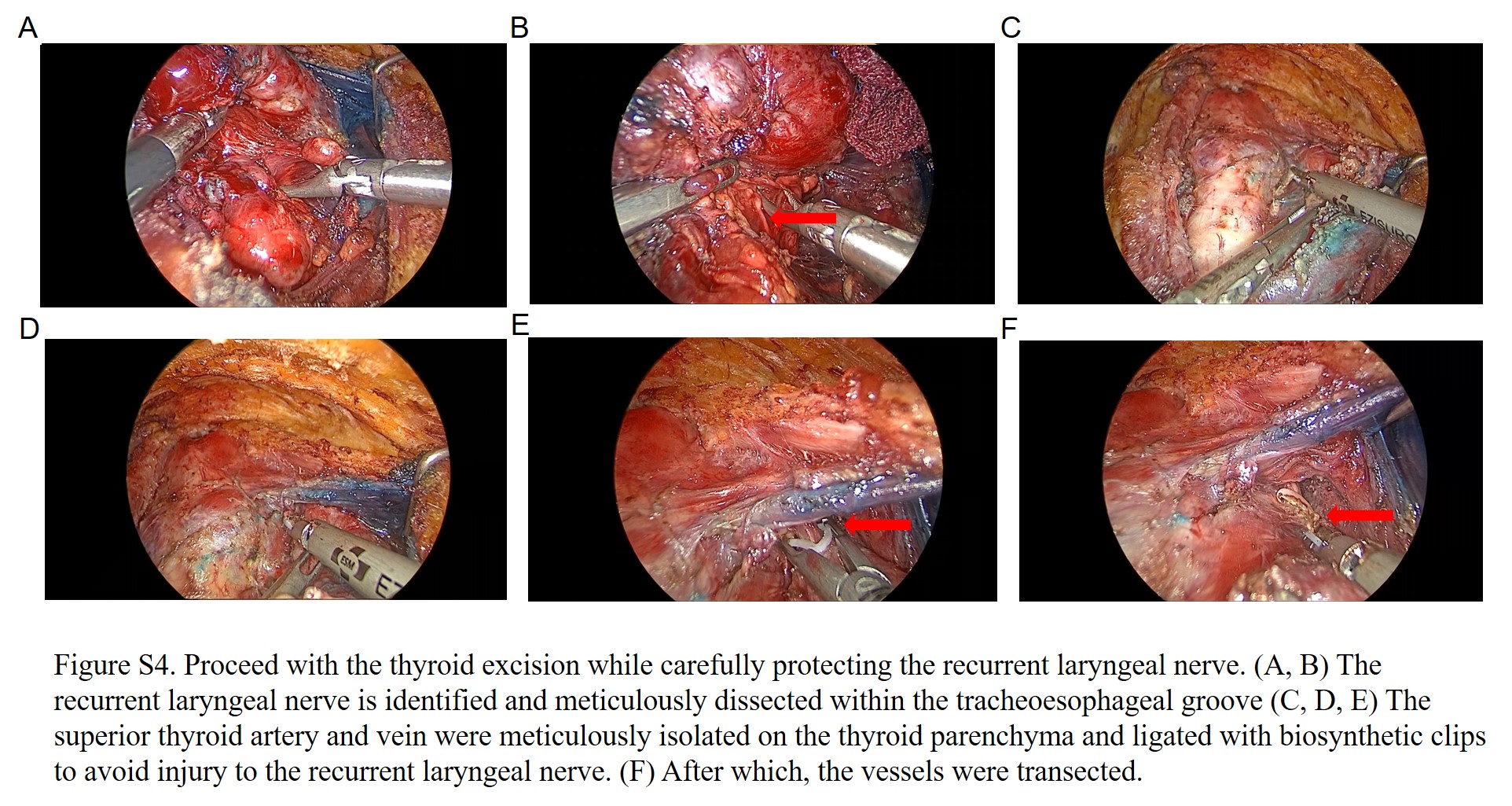

Supplement: Supplementary file 4 [file Image4.jpeg]

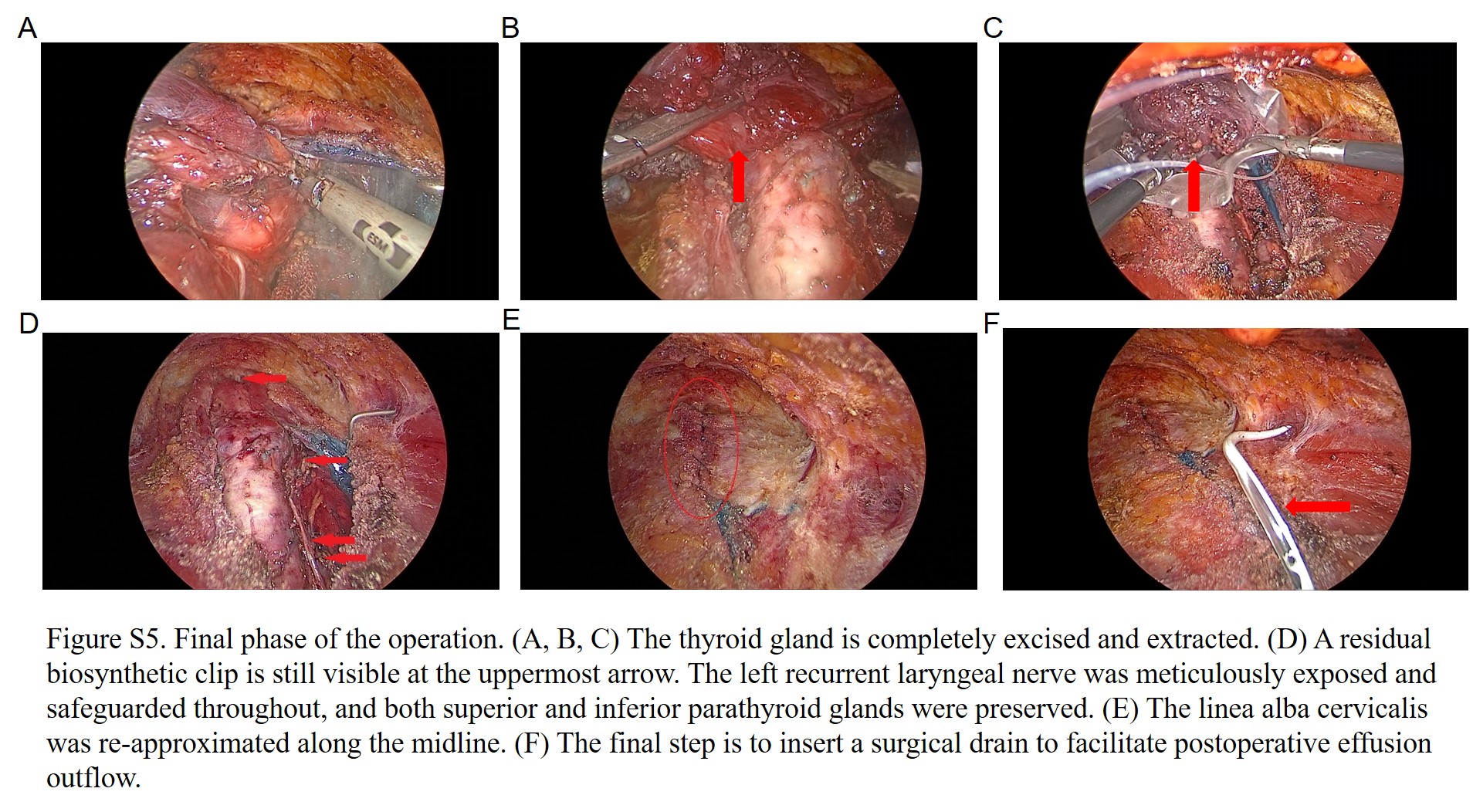

Supplement: Supplementary file 5 [file Image5.jpeg]
